# Embodiment of Wearable Technology: Qualitative Longitudinal Study

**DOI:** 10.2196/16973

**Published:** 2020-11-03

**Authors:** Elizabeth C Nelson, Anneke M Sools, Miriam M R Vollenbroek-Hutten, Tibert Verhagen, Matthijs L Noordzij

**Affiliations:** 1 Faculty of Electrical Engineering, Mathematics and Computer Science University of Twente Enschede Netherlands; 2 Department of Psychology, Health and Technology University of Twente Enschede Netherlands; 3 Ziekenhuis Groep Twente Almelo Netherlands; 4 Center for Market Insights Amsterdam University of Applied Sciences Amsterdam Netherlands

**Keywords:** wearability, implantable wearable, body extension, smart prosthesis, implantable devices, technology dependence, cognitive prosthesis, phenomenology, embodied self-discrepancy, technology addiction, longitudinal qualitative design

## Abstract

**Background:**

Current technology innovations, such as wearables, have caused surprising reactions and feelings of deep connection to devices. Some researchers are calling mobile and wearable technologies cognitive prostheses, which are intrinsically connected to individuals as if they are part of the body, similar to a physical prosthesis. Additionally, while several studies have been performed on the phenomenology of receiving and wearing a physical prosthesis, it is unknown whether similar subjective experiences arise with technology.

**Objective:**

In one of the first qualitative studies to track wearables in a longitudinal investigation, we explore whether a wearable can be embodied similar to a physical prosthesis. We hoped to gain insights and compare the phases of embodiment (ie, initial adjustment to the prosthesis) and the psychological responses (ie, accept the prosthesis as part of their body) between wearables and limb prostheses. This approach allowed us to find out whether this pattern was part of a cyclical (ie, period of different usage intensity) or asymptotic (ie, abandonment of the technology) pattern.

**Methods:**

We adapted a limb prosthesis methodological framework to be applied to wearables and conducted semistructured interviews over a span of several months to assess if, how, and to what extent individuals come to embody wearables similar to prosthetic devices. Twelve individuals wore fitness trackers for 9 months, during which time interviews were conducted in the following three phases: after 3 months, after 6 months, and at the end of the study after 9 months. A deductive thematic analysis based on Murray’s work was combined with an inductive approach in which new themes were discovered.

**Results:**

Overall, the individuals experienced technology embodiment similar to limb embodiment in terms of adjustment, wearability, awareness, and body extension. Furthermore, we discovered two additional themes of engagement/reengagement and comparison to another device or person. Interestingly, many participants experienced a rarely reported phenomenon in longitudinal studies where the feedback from the device was counterintuitive to their own beliefs. This created a blurring of self-perception and a dilemma of “whom” to believe, the machine or one’s self.

**Conclusions:**

There are many similarities between the embodiment of a limb prosthesis and a wearable. The large overlap between limb and wearable embodiment would suggest that insights from physical prostheses can be applied to wearables and vice versa. This is especially interesting as we are seeing the traditionally “dumb” body prosthesis becoming smarter and thus a natural merging of technology and body. Future longitudinal studies could focus on the dilemma people might experience of whether to believe the information of the device over their own thoughts and feelings. These studies might take into account constructs, such as technology reliance, autonomy, and levels of self-awareness.

## Introduction

Individuals are increasingly wearing devices on their bodies, which monitor their behavior and provide coaching through associated apps. On one hand, the interaction with these types of devices might result in a sustained experience, where technology extends the body [1-3], cognition, and even self, which was recently conceptualized as wearable technology embodiment [4]. This is in line with other work in which wearables were referred to as cognitive prostheses [5,6]. With this label, the positive enabling effects of the wearable on cognition (eg, improved self-regulation for going to bed on time with sleep tracking) is put in the same category as those that can be expected in the motoric domain from a physical prosthesis. On the other hand, some researchers have suggested that the effects and even the active use of wearables are short lived (ie, not more than a few weeks) and inconsequential [7]. This might also be in line with a popular belief that wearables, such as an activity tracker that counts and displays steps, are devices that might have an initial appeal, but will quickly be discarded after the novelty effect wears off [7-11]. Importantly, it is unknown how people experience wearables over longer periods of time given the dearth of longitudinal qualitative research on this topic [8,12,13]. What is known from a qualitative and longitudinal perspective is how people engage with, adapt to, and embody physical prostheses [14-16]. Given the initial viewpoint presented above, the expectation for wearables is that there may be noteworthy overlap between user experiences with and perspectives on prosthesis and wearable embodiment. However, if wearables in practice are quickly discarded, the alignment with physical prosthesis embodiment (on which people rely heavily to regain physical capabilities) will be problematic or even impossible. The aim of this research was to study to what extent the experiences that previously have been associated with adaptation over several months to a physical prosthesis [14] are applicable and representative for the embodiment of a wearable.

Murray [14] investigated user perspectives on the embodiment of a physical prosthesis, which provided important insights into the phases of embodiment (ie, initial adjustment to the prosthesis) and the psychological responses to the prosthesis (ie, accepting the prosthesis as part of their body). The process phases and psychological responses represent a comprehensive foundation to understand and categorize embodiment of not only a prosthesis but also any item embodied by the user. Murray’s six themes include (1) adjustment to a prosthesis; (2) balance of the body; (3) awareness of the prosthesis; (4) the prosthesis as a tool or corporeal structure; (5) the knowing body; and (6) the phantom becomes the prosthesis, extending the body.

The first theme (adjustment to a prosthesis) describes the initial period of mental and physical adjustment after receiving a new prosthetic device. In order to embody a prosthesis, an individual must create a working relationship with it, integrating it into the daily routine. The maintenance of the prosthesis during this time is described as considerable, but the tasks are eventually absorbed into a schedule or rhythm requiring little thought. Past research on technology use describes a similar process during the initial period with a new technology device. Research has found that interaction with a new technology first evokes a cognitive response to the device, followed by a behavioral response [17,18], which can either lead to the adoption or abandonment of the technology [19]. It is unknown to what extent initial technology adoption and experience is similar to physical prosthesis embodiment during the first 3 months of active use.

The second theme from Murray (the balance of the body) evaluates the adjustment to the imbalance created by the amputation or prosthetic device. Balance is key in creating a good fit between an individual and a prosthesis, making it easier to wear. Individuals wearing a well-balanced prosthetic device describe an ongoing process of “subconscious compensation” to naturally reposition the body to improve balance [14]. Creating a good fit with a prosthetic limb relates closely to the concept of wearability, which involves the degree of physical, mental, and/or social comfort in wearing a device [20]. Highly wearable technologies are comfortable and easy to wear (no distraction or attention demand) [20] and have been shown to have higher success rates for continued use [21]. Patients receiving a new wearable to replace an older version described feeling like “a living medical instrument” [22] while wearing the device. What is interesting to discover is to what extent the wearability of the wearable device impacts the embodiment of the device.

The third theme from Murray (awareness of the prosthesis) explains the changing nature of use over time (ie, disturbances or ease of use). A prosthesis that is embodied is integrated into the body, operating automatically without disruption or attention. Feedback from the technology, or contextual awareness [20], also has to feel automatic. Therefore, push notifications or other content can either be welcomed or be considered a disruption. The quality of the information and the extent to which people are engaged can ultimately impact their behavior [23,24]. Unpredictable or unusual feedback can engage people and compel their attention [20]. This presents a possible contrast between the unwelcomed interruption or awareness of a physical prosthesis and the welcome feedback interruption of a wearable.

Murray’s fourth, fifth, and sixth themes analyze the deeper emotional relationship with the technology. Murray’s fourth theme (the prosthesis as a tool or corporeal structure) examines whether individuals felt either a sense of completeness with the device or the prosthesis remained a helpful but external tool [14,25-28]. Research in mobile phones focuses on the adoption of technology beyond a technical tool, with features such as gamification [29,30]. Gamified elements have been successful in short-term analysis [31-33] but have been critiqued by some for their questionable ability to aid in long-term goal attainment and behavior change [29]. Murray’s fifth theme (the knowing body) involves the body’s feedback to the mind. This can include the body’s muscle memory where the feedback of an embodied prosthesis can be considered the same as the rest of the body. Murray’s sixth theme (the phantom becomes the prosthesis, extending the body) explains where the prosthetic limb is considered as part of the body, possibly replacing the experience of a phantom limb (when the missing limb is felt). Interestingly, prosthesis research has shown that a phantom limb and a prosthesis can intertwine in an individual’s mind, merging into one perceived entity [14]. All three themes examine the integral connection of the brain to the prosthesis. Research has suggested that technology may interlace with our minds to take over some tasks, such as navigation [34], and take on new ones, such as quantifying sleep and activity [4,26]. Clark [35] argued that cell phones were not simply technological tools but upgrades of the mind. Could the digital feedback intertwine with individuals’ perceptions of their sleep or activity creating a combined feedback experience similar to the intertwining of a prosthesis and phantom limb? Additionally, what are the implications when it does?

While certain similarities have been established between wearable and physical prosthesis experiences, there are no longitudinal studies of wearables that can be compared with the prosthesis experience. Adapting the themes of Murray [14] to wearables can shed light on how a wearable may be embodied similar to a physical prosthesis in terms of both the phases of embodiment (ie, initial adjustment) and psychological responses (ie, accepting it as part of their body). This study extends current research to show usage patterns of a wearable over the long-term.

## Methods

### Participants

**Figure 1:**
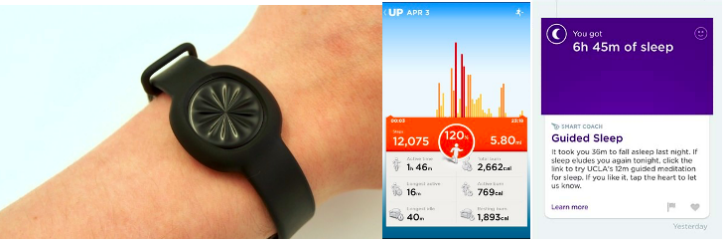
Jawbone UP Move accelerometer and mobile phone app.

Over a 9-month period, a sample of 43 employees out of 400 from a large consultancy company in Amsterdam, The Netherlands, wore wearables, specifically the Jawbone UP Move, which consists of a wristband and mobile app, as well as a web platform with login (Figure 1) [36]. The wristband lit up and vibrated when a goal was met but did not have an interface or connection to any function or information beyond activity and sleep (ie, email, navigation, and phone calls). The mobile app included activity for the current day (including calories burned, idle time, distance traveled, and floors climbed) and time slept the previous night (including minutes asleep, minutes awake, and deep sleep and light sleep quality). The participants were part of a larger work wellness program and longitudinal research study. The wellness study was put together to experiment with improving health in the office and included aspects such as yoga classes, increased distribution of plants, and nutritious food. No incentives were provided, and there was no supervision of the group. People were not reminded about using the devices and could leave the study at any time.

Of the 43 participants, 12 (mean age 35 years, SD 6.5 years, range 25-50 years; five female and seven male participants) volunteered to be part of this qualitative study. The wristbands were worn continuously by the participants and measured their sleep, activity, and inactivity. The mobile app was accessible on their smartphones and also over the internet with their personal login. Daily summaries were provided on the mobile app (Figure 1). Gamification elements in the app included duels (daily activity competitions) and daily goals of sleep and activity. Duels could be sent to other individuals in the workplace group to compete for the highest step count that day. The wristband vibrated and lit up when the daily activity goal was reached. Reminders were sent few (two to three) times per day regarding activity progress via the app. Data were transferred automatically from the wristband via Bluetooth to the mobile app and thus worked offline. Two of the male participants had worn a fitness tracker prior to the study. A signed consent form was collected at the beginning of the study, and verbal consent was obtained at the start of each interview.

### Interviews

Participants were invited to the study via email. The series of semistructured interviews followed Murray’s [14] method of interviewing three times over the course of 9 months. While the main questions remained the same over the three interviews, some probing was done to dive deeper into participants initial responses to questions. The interviews were conducted around 3 months, 6 months, and 9 months after the participants started wearing the wristbands. The final interview was conducted 6 to 8 weeks after the work wellness program concluded. A pretest was conducted with four individuals from the study for comprehensiveness and clarity of the wording of the questions [37,38]. Minor changes were made to the wording of the questions based on feedback from the pretest for better comprehension or to stimulate a more thorough answer.

At the start of each interview, participants provided consent for the interview to be recorded. The interviewer explained to the participants that their responses would remain anonymous and would be used only for scientific publication. The participants were interviewed face to face with the exception of one phone call. Interviews were recorded and then transcribed into documents and coded according to participant (code name) and interview number (ie, interview 1, 2, or 3) [37]. Participants were Dutch natives and spoke English fluently. All interviews were conducted in English face to face with one member of the research team. No prior relationship between the interviewee and interviewer existed before the interviews began. Interviews were conducted in a quiet section of the office, and the duration ranged from 8 to 35 minutes depending on the participant’s response length. Most participants responded openly and did not require much probing.

### Question Adaptation

The prosthesis-based themes and questions from Murray’s [14] research were adopted for use in this wearable technology study (Table 1 and Multimedia Appendix 1). Some themes were combined. The “adjustment to the prosthesis” and the “tool or corporal structure” themes were combined into one theme. The gamified tool was too intertwined with the initial process of adjusting to the prosthesis. The “knowing body” and the “phantom becomes the prosthesis” themes were also combined. While the differences between the knowing body and the phantom becomes the prosthesis themes are clear when applied to a physical prosthesis (ie, the mind and the body), the overlaps when applied to a wearable make it difficult to separate the two because both themes are in relation to the mind. An additional question was added to see how individuals compared using the wearable to their smartphone.

**Table 1:** Original themes of Murray and adapted themes with descriptions.

Original theme by Murray [14]	Description	Adapted theme	Description
Adjustment to the prothesis Tool or corporal structure	Becoming familiar with a prosthetic device for the first time, and physical and psychological adjustment. Experiencing the prosthetic device as either a tool or part of the body.	Adjustment to the wearable	Adjustment to the gamified tool/wearable during the initial period (months 1-3) with the device.
The balance of the body	Body weight distribution and balance.	Wearability	Level of comfort or ease of wear.
Awareness of the prosthetic device	The attention and awareness that was given to prosthesis use.	Awareness of the wearable	Level of awareness of the wearable and whether the aspects demanding awareness are welcome or disruptive.
The knowing body The phantom becomes the prosthesis, extending the body	The body’s feedback to the mind (including the prosthetic body part). A prosthetic limb being experienced as part of the body.	The embodied wearable extending the mind	Experiencing the information as part of cognition and feeling and/or believing the information is as valid or more valid than subjective experience. Experiencing the device as part of the body.

### Procedure and Analysis

In order to analyze the participant interviews, participants were given a pseudonym. Responses were then given the pseudonym as well as the interview sequence (in this case, interview 1, 2, or 3). ATLAS.ti (Scientific Software Development GmbH) and Microsoft Excel (Microsoft Corp) were used to analyze the responses for themes and longitudinal trends. Both inductive and deductive reasoning were used. Responses were coded into one or more of the adapted themes of Murray [14]. To stay open to additional themes, a constant comparison method [39,40], a form of thematic analysis [41], was utilized throughout the series of interviews to understand how participants experienced the wearable. This particular form of qualitative analysis was selected because its approach is useful in shedding light on how individuals experience the technology over the long term [14,38]. This dual process of constant comparison allowed us to code responses based on predefined categories, as well as discover themes or categories from the data itself.

## Results

### Themes

During analysis, two additional themes were discovered and included. A high number of responses were recognized relating to engagement/reengagement and comparison to another person or device. No additional questions were added to the semistructured interviews. While past studies have compared new prostheses to old prostheses, it was not a theme of Murray [14]. We believed these discovered themes do shed light on the nuances of wearable technology embodiment not yet discovered. The interrater reliability was calculated for all the themes using Cohen κ (Multimedia Appendix 2), with a 10% sample of the responses. Two researchers (ECN and MLN) reached a Pr(a) of 0.84 for all themes (Multimedia Appendix 2) after three rounds of revisions and thus a strong level of consistency [42]. It was established that the data collection did reach saturation.

### Theme 1: Adjustment to the Wearable

During the initial 2-month period with the wearable, the participants described near constant interaction with the technology, checking it multiple times per day and sometimes only a few minutes apart to see how many steps had been achieved. Participants reported that the first months were “very motivating” and could be attributed to being drawn into the gamified elements of the technology. Most of the participants were experiencing a wearable for the first time (10 out of 12). Many individuals reported that they started walking to other parts of the office to have “face-to-face check-ins instead of sending a text or email.” The technology also awarded digital badges to participants who challenged other participants to a “duel” and logged the most steps that day. However, these challenges or duels were reported to greatly decrease over time. All participants reported changes in their behavior and in making decisions to try to get more steps and/or to sleep longer or more deeply because of such aspects as the daily goal. One participant responded as follows:

I think in the beginning it's more of a high that you really want to achieve 10,000 steps.

Eva; first interview

While most of the responses were positive, there were some negative reactions to the recommended daily goals. The app suggested 10,000 steps and 8 hours of sleep. The suggested activity goal was explained as an “exciting challenge” in the beginning but proved to be “quite difficult” during weekdays, causing frustration and sometimes demotivation. Forgetting the wearable was also reported as frustrating especially during active days that could have increased the weekly average. In the first interview, Nate stated that he took 3000 steps on a normal weekday at the office. He expressed frustration with having to do more than three times that number to reach his daily goal. He made the following statement:

That's really crazy then it's difficult to get your 10k steps which I did as my target but it's really difficult to get there and I don't think you can get there with a normal job.

Nate; second interview

This initial adjustment time was also when the participants stated they “set up a routine” with the device, including pressing the button after waking in the morning and before sleep at night, learning the number of steps in typical activities, such as walking to work and usual errands, and checking step count throughout the day. Murray described this period as a time of acceptance or rejection when the individual and the prosthetic device must synchronize to achieve a working partnership. The participants reported a range of feelings to the new device such as feeling “familiarity,” “curiosity,” and “adjustment.” These feelings show a similar pattern to prosthesis embodiment and suggest that the devices are embodied or are in the process of embodiment.

### Theme 2: Engagement/Reengagement

While the first period of adjustment was also the period of most frequent use for all participants (n=12), overall, we saw a process of engaging, disengaging, and reengaging over the long term. Over the 9 months, most participants experienced at least two distinct periods of heavy use and two periods of infrequent use. One participant commented as follows:

I was very curious. How does it work and how much do I walk and now it became just a part of the day.

Eliz; first interview

Other participants described “missing the technology” when not wearing it, and a sense of “starting over” when the technology was not used for a period of time. Matthew described a period of infrequent use and had experienced sleep deprivation and reduced activity. This period was during a time of intense workload and long hours. He described the challenge of reintegrating the device into his daily routine again, where increased use helped him to regulate his routine once again. His comment was as follows:

Using the device again it feels like some kind of start over.

Matthew; second interview

One male participant in the first interview reported being especially interested in the sleep patterns and the quality of sleep, putting much focus on it. It was something that he had “not focused on” before getting the device. By the third interview, he believed he had learned to measure his sleep independent of the device. Interestingly, when participants were asked if they preferred the technology quantifying their activity and sleep or desired to gain the skill of knowing their quantified health data, most (n=9) preferred to continue using the technology. The reengagement by all participants and desire to continue using the technology as opposed to gaining the skill suggests a certain level of embodiment of the device.

### Theme 3: Wearability

The wearable in this study was quite small compared with others on the market, so neither women nor men complained about its bulkiness. In general, participants in this study described the device as “comfortable.” The participants received the device in the autumn and initially talked about adjusting to sleeping with it, but most (n=10) said they “did not notice” the wearable or that it “did not bother them.” One participant chose not to sleep with the wearable on finding it uncomfortable after a few nights. When the spring and summer months came, some participants (n=2) reported that the device became “itchy” during high heat. A participant who also struggled initially with discomfort at night reported added discomfort during the summer in high heat. Interestingly, many participants described the experience similar to wearing a watch and stated that the device was “hardly noticeable.” Yet, most participants did not wear a watch and had not used a watch for many years. No participant stopped wearing the wearable completely owing to comfort issues. One participant made the following statement:

It felt like I was wearing a watch. In winter, it was ok but in summer I thought it was sometimes a bit annoying.

Mary; third interview

The level of perceived attractiveness seemed to add to the ease of wear. Most participants (n=10) found the device attractive, and many (n=9) also enjoyed being asked what the device was. This positive attention and perceived attractiveness made the device quite wearable. Interestingly, by the end of the study, many participants stated a desire for their next wearable to have “more functionality” and a different look (n=9), and to be more like a smartwatch than an activity tracker. One participant made the following statement:

I don’t really feel it. It doesn’t bother me at all. My only thing is that it's ugly.

Mathew; first interview

Wearability thus seemed to focus heavily on seasonality, the size and feeling of a watch, discomfort during sleep or high heat, and the level of perceived attractiveness.

### Theme 4: Awareness of the Wearable

After the initial 2 months, many participants interacted much less with the device. The daily results and content from the mobile app (Figure 1) were explained as “repetitive” and “did not change or surprise” the participants anymore. John initially reported being “addicted to the device” and did not experience the first low interaction period until much later than the other participants. However, by the third interview, he described a feeling of “boredom with the content” of the mobile app. The majority of the participants did not have a high level of awareness of the device stating it was “just there.” John made the following statements:

You were constantly looking how many steps I've taken in the last 10 minutes and now it's become like I said hardly think of that it's there… you're being reminded because people keep asking what's that on your wrist. But not because I feel it, sense it. It's just there.

John; first interview

There are also some notification or suggestions for what you can do you just can't help reading them so when they pop up you see them and obviously see there is a pattern or there are certain standard suggestions once you've seen them.

John; second interview

All participants reported checking sleep and activity once in the morning for sleep and at the end of the day for activity after the initial period of high use. The wearables required a button to be pushed before bed and when awake in the morning as an extra framework to accurately measure sleep time. The wearable did not demand attention similar to a well-fit prosthesis, but the feedback was considered boring (ie, low contextual awareness).

### Theme 5: The Embodied Wearable, Extending the Mind

Many participants expressed “intense” reactions to or relationships with their device during the 9-month study and described “missing the device” when it was forgotten. While some reported experiences of “addiction,” at minimum, reports referred to the technology as likely to be “habit forming.” Four participants said the device felt like part of their body. Some participants made the following statements:

There won’t be many moments that I forget about it or won’t wear it. I take it to the gym it's really part of my body.

Anna; second interview

I'm quite surprised that it's become such an automatic, almost part of your body so to say. Like I said I don't feel it, I don't notice it, so it's there.

John; first interview

In the second interview, John described what he may do moving forward. He might either stop using the device or keep it for his physical training to “keep himself sharp.” By the third interview, the wearable was replaced with another focused on running training, which was a gift from his team. He enjoyed the additional information like heart rate. In Tom’s first interview, he described feeling powerless and wanting to know his biological data when he was without the device. He stated “It’s a crazy feeling when it’s off.” He restated this in the second interview and mentioned “because you get comfortable wearing it, so when you take it off you miss it.” In the third interview, he seemed to refer to himself and the technology working together.

Many participants referenced the calming effects confirming health behavior but could not answer why. One participant stated in all three interviews that it was comforting knowing what the activity had been. This was true for reaching milestones, such as 10,000 steps, and when the device confirmed feeling tired, such as after a bad night’s sleep. The adverse reaction existed when goals were not met and feelings of discomfort or frustrations arose. One participant commented as follows:

Your feeling is being confirmed. If you think it was a rough night then you look at the app “oh it was”.

Eliz; second interview

All participants experienced “surprising” and “discomforting” feedback regarding their sleep and/or activity. Some participants (n=4) reported a shift later in the study and started questioning the technology as an “accurate/correct measurement” while continuing to use it. They seemed to be struggling to decide whether to believe themselves or the device. Interestingly, none of the four participants reached a final decision on “whom” to trust by the end of the study. The indecision and willingness to trust the wearable above one’s own feeling would suggest some embodiment had taken place.

### Theme 6: Comparison to Another Device or Person

A new theme was discovered based on comparisons to other devices. This theme was further explored using responses from the adapted questions, such as how the wearable compared with the smartphone. Half of the participants (n=6) found the experience similar to receiving their first smartphone and checking their wearable “automatically” and with “little thought,” although this statement was not consistent for individuals over the three interviews (Multimedia Appendix 3). This was explained by the fact that the wearable, like the phone, was “constantly with them,” gave “updated data,” and was something they “checked often.” The experience was also compared to that of a singular mobile app on a phone but was felt to be less relevant than the full functionality of a smartphone and was therefore less frequently used. This was believed to be a reaction to the evolution of technology and the initial experience of exploration with a new device. One participant made the following statement:

It’s not like my phone. It’s a pull it's not addictive and disappointment is maybe not the correct word. It's more like you want to wear it because you have it and it doesn't take you a lot of effort to have it record all the steps.

Tom; third interview

One group of participants (n=8) had a reliance on the technology for activity and sleep evaluation, which they explained as similar to a smartphone. While most participants found they could not know their sleep and activity without the device, they reported at least some level of learning to gauge activity and sleep. The wearable took the place of a guide, even being referenced to as a “mother” because of the reminders to be healthier. One participant made the following statement:

Every morning you get the alert about the notification of your sleep. It's good to see and I track how much is my sleep and is it long enough sound sleep. I want to try more sound sleep than light sleep because I sleep all night but more lite than sound sleep. But it’s funny, I call the notification to go to sleep ‘mother’.

Matthew; second interview

Furthermore, most of our participants stated that they depended on their phone for navigation (n=11) and phone numbers (n=12) [43]. In the first interview, one participant stated that there was “no way of realistically guessing progress.” By the third interview, the participant stated that it may be possible to guess progress after using the device for so many months, but still felt the device was more accurate and motivating.

## Discussion

### Principal Findings

This study is one of the first to track the use of wearables in a longitudinal qualitative study, providing a nuanced and varied insight into how this technology is used, embodied, and integrated into people’s daily life. In this study, we found that although previous short-term research seemed to suggest wearables are quickly abandoned [7,8,10,11], there were alternating periods of engagement, disengagement, and reengagement over a 9-month timeframe. The fluctuating engagement is consistent with Oliver’s [44] belief that the usage process of a device includes a sequence of acceptance, experience, verification, and continued use. On further reflection, it is understandable that engagement and reengagement did not come up in research on prosthetic limbs. Limb prostheses are associated with an experience of acceptance of the prosthetic device but rarely periods of reengagement, because this would mean giving up the regained functions. Furthermore, a limb would not likely be compared to another person.

It is also important to note that unlike the report by Murray [14], our participants were not obliged to wear the wearable, yet none of the participants chose to leave the study. Many participants expressed desires for higher functioning devices as their next wearable, suggesting a continued interest in the device/process. The most frequent use was experienced by all participants at the beginning of the study. This was during the period where many described getting the device into their daily routine and enjoying the gamified elements of the device, including competition with friends or colleagues. This high level of motivation is experienced often with technology adoption [17], especially technology including gamification [21], and is explained by theoretical paradigms, such as the innovation diffusion theory, which describes how beliefs, such as relative advantage, influence how individuals decide to adopt a technology [45,46].

We also discovered differences and similarities between a wearable and a limb prosthesis. With both, successful adjustment to the device during the first period and wearability are key to adoption and embodiment. High wearability means the device should be comfortable and should integrate into the body to fit within the individual’s overall functioning without constraining any motion [47]. The wearable we chose was relatively small, and if it had been larger or heavier, or had hindered movement, the embodiment could have been less successful [48]. Itchiness in warmer seasons did seem to provide some discomfort. Wearability is especially important for wearables that are an optional addition to the body, while a limb prosthesis is considered a needed replacement. The participants’ positive reactions to public approval are supported by the Theory of Planned Behavior [49]. Initial reactions to the devices also suggest some form of empowerment or perceived behavioral control [21]. Subjective norms can pressure individuals for approval, changing their behavior or perceptions. Wearing a device that received positive attention could reinforce the choice to wear the device [48] and perceive the device as more wearable due to the emotional benefits. Wearables also differed from Murray’s [14] prosthesis research in terms of awareness. The wearable did not create bothersome awareness similar to a well-fit prosthesis but was not considered engaging in the long term (ie, low contextual awareness).

The feedback from the wearable created an interesting dilemma. Adding a quantified measurement of sleep and activity to the perceptions of sleep and activity created parallel feedback that could be either confirming (ie, confirm a good or bad night’s sleep) or invalidating (ie, present information radically different than experiential perceptions). Parallel feedback can create a fracture in the sense of self and can lead to either distrusting the device, one’s self, or both [50]. When the individual trusts the information coming from the device, this can manifest in self-regulating responses. However, when the information does not match the individual’s beliefs, this can cause a reaction of self-discrepancy, where the individual holds simultaneous yet incompatible self-beliefs possibly causing distress [51]. A merging experience has been described in technology embodiment research [2,3] and cyborg intentionality where the person and technology both come with separate intentions (mediated intentionality/composite intentionality) combined into an experience [1]. For instance, the wearable did not intend to be a “mother figure,” yet the combination of individuals’ experiences with the wearable’s intention created that “human dynamic.” We did not find that participants experienced the discrepancy as particularly distressing, but awareness of the discrepancy seemed to increase over time. Interestingly, individuals more boldly expressed the inconsistency as time went on, but these individuals continued to struggle with whether to abandon the device. There were clear individual differences in the extent to which people experienced discrepancies and the willingness to put more trust in the technologically mediated information than in subjective experience. The experience of seeing the device as a “mother figure” may show both negative and positive reactions to self-regulation. Further longitudinal investigation of this phenomenon has been called for to see how real-time monitoring of human functions and constant presentation of the data influence self-perception [50]. The self-discrepancy theory has not yet been extended to embodiment literature. We believe this research provides the first evidence that the self-discrepancy theory is applicable to embodiment phenomena.

In addition to the adapted themes of Murray [14], we discovered that wearables do have similarities to other devices such as mobile phones. We see increasing dependence on technologies, such as mobile phones and navigation technologies, that extend or replace our cognitive efforts and competencies (ie, remembered phone numbers and navigation) [6,34,35]. Research in mobile addiction or “smartphone dependence” has addressed its effect on mental health issues with regard to compulsive usage [52]. Our comparison of wearables to initial smartphone experiences suggests that wearables could have similar reactions to the technology, especially as the technology innovates. Research has found that individuals can create an emotional attachment to their mobile phones [53], and literature within mobile technology interaction has proven that individuals can believe their phones are extensions of themselves [4,8,54,55].

### Limitations and Future Research

This study has few limitations. First, the study included a relatively small and homogenous group (n=12) that was repeatedly measured over a period of 9 months, making further generalization a question for future research. The participant group was representative of a consumer group (young to middle-aged highly educated professionals) interested in wearables. For many of the participants, this was their first time using wearables, which provides great insights into the experiences of first-time users. However, we recommend performing a further study on participants having ongoing experience with wearables and mobile devices. The wearables were small and unobtrusive, and while this helped us to see the experience of a highly wearable device, we acknowledge that not all wearables are unobtrusive. Additionally, while all participants could abandon the study at any point, they were part of a wellness program at work, which may have encouraged them to continue. Our discovered theme of comparison to another device or person could indicate levels of technology dependence similar to mobile phones. We recommend further research on technology dependence and addiction to various types of technologies. Furthermore, this study did not examine or provide explicit information to participants about the validity and reliability of the consumer wearables. This is an important research topic in and of itself [56], and for this study, the participants were referred to the disclaimer of the company indicating the device was not a medical grade device and was only intended for lifestyle monitoring and suggestions. We also acknowledge the critique on phenomenological studies more generally [57] that language mediates experience, and hence, a direct window on the experience itself was not our aim. Finally, this study compared a typical “dumb” physical prosthesis to a “smart” wearable, which are two areas already merging into a shared space. This research provides important insights into the experience of future “smart” prostheses and technologies already embodied by our bodies and minds [4].

## Appendix

Multimedia Appendix 1 Interview questions.

Multimedia Appendix 2 Cohen kappa.

Multimedia Appendix 3 Comparisons.
