# Supplementary material for: Embodiment of Wearable Technology: Qualitative Longitudinal Study
Source: JMIR Mhealth Uhealth. 2020 Nov 3;8(11):e16973. doi: 10.2196/16973 (PMC7671844; doi:10.2196/16973)
Supplement: Multimedia Appendix 1 [file mhealth_v8i11e16973_app1.docx]

**Multimedia Appendix 1.** Interview questions.

| Murray 2004 | Adaptation |
| --- | --- |
| What sensory feedback do you get from your prosthesis? | What feedback do you get from your jawbone UP wristband and app? |
| How closely do you have to attend to what you are doing with your prosthesis? | How closely do you have to attend/pay attention to what you are doing with your jawbone UP? |
| In what ways are you reminded of your prosthesis? | In what ways are you reminded of your jawbone UP? |
| How does your experience of using your prosthesis now differ to when you first began using one? | How does your experience of using your Jawbone UP now differ to when you first began using one? |
| Do you think that you use your prosthesis in a similar manner to your anatomical limb, that is, with little thought? | Do you think that you use your jawbone UP in a similar manner to your (phone), that is, with little thought? |
| Have you/do you experience a phantom limb? Can you tell me about these experiences? | How do you feel when you are without your wristband or app or can't see your progress? Do you think you could gage your progress without your jawbone UP? |
|  | Last question of last interview: To when you first got your smart phone what extent to do you feel this is different? |
