# Supplementary material for: Embodiment of Wearable Technology: Qualitative Longitudinal Study
Source: JMIR Mhealth Uhealth. 2020 Nov 3;8(11):e16973. doi: 10.2196/16973 (PMC7671844; doi:10.2196/16973)
Supplement: Multimedia Appendix 2 [file mhealth_v8i11e16973_app2.docx]

**Multimedia Appendix 2.** Cohen's Kappa.

|  | | | |  |  | Researcher 2 | | |  | |  | |  | | | |  |  |
| --- | --- | --- | --- | --- | --- | --- | --- | --- | --- | --- | --- | --- | --- | --- | --- | --- | --- | --- |
|  | |  | Theme 1 | | Theme 2 | Theme 3 | | Theme 4 | | Theme 5 | | Theme 6 | |  |  |  |  |  |
| Researcher 1 | | Theme 1 | 1 | | 1 |  | |  | | 1 | |  | | 3 | 0.23 |  |  |  |
|  | | Theme 2 | |  | 2 |  |  | |  | |  | | 2 | | 0.15 |  |  |  |
|  | | Theme 3 | |  |  | 2 |  | |  | |  | | 2 | | 0.15 |  |  |  |
|  | | Theme 4 | |  |  |  | 2 | |  | |  | | 2 | | 0.15 |  |  |  |
|  | | Theme 5 | |  |  |  |  | | 2 | |  | | 2 | | 0.15 |  |  |  |
|  | | Theme 6 | |  |  |  |  | |  | | 2 | | 2 | | 0.15 |  |  |  |
|  | | Total | | 1 | 3 | 2 | 2 | | 3 | | 2 | | 13 | |  |  |  |  |
|  | |  | | 0.08 | 0.23 | 0.15 | 0.15 | | 0.23 | | 0.15 | |  | |  |  |  |  |
|  | | Agreement | | 1 | 2 | 2 | 2 | | 2 | | 2 | | 11 | |  |  |  |  |
|  | |  | |  |  |  |  | |  | |  | |  | |  |  |  |  |
|  |  |  |  |  |  |  |  |  |  |  |  |  |  |  |  |  |  |  |

| Pr(a) | 0.84 |
| --- | --- |
| Pr(e) | 3.48E-05 |
| k | 0.846E-01 |
