# Supplementary material for: Embodiment of Wearable Technology: Qualitative Longitudinal Study
Source: JMIR Mhealth Uhealth. 2020 Nov 3;8(11):e16973. doi: 10.2196/16973 (PMC7671844; doi:10.2196/16973)
Supplement: Multimedia Appendix 3 [file mhealth_v8i11e16973_app3.docx]

**Multimedia Appendix 3** Comparisons.

| Theme & Participant Pseudonym | Interview 1 | Interview 2 | Interview 3 |
| --- | --- | --- | --- |
| Adjustment |  |  |  |
| Anna | High focus to improving sleep and activity (also diet) | X | X |
| John | Looking at it constantly | X | X |
| Matthew | Learning curve to get in routine | X | X |
| Tom | Became part of routine | X | X |
| Mary | Adjusting to step goals | X | X |
| Eliz | Part of my ritual | X | X |
| Luke | Constant use in getting into routine | X | X |
| Eva | Adjusting to new routine | X | X |
| Nate | Adjusting to pushing button before after sleep | X | X |
| Sam | Changing behavior | X | X |
| Tristen | Adjusting to improve sleep | X | X |
| Broderick | Integrating into day | X | X |
| Engagement/reengagement |  |  |  |
| Anna | Highly engaged after wristband was broken a few days | Engaged | Engaged after a period of disengagement |
| John | Highly engaged | Engaged | Engaged after a period of disengagement |
| Matthew | Highly engaged | Start over | Reengaged |
| Tom | Highly engaged- loves “duels” with colleagues | Engaged | Very Engaged |
| Mary | Highly Engaged | Disengaged, losing attention | Not using after a period of Reengagement |
| Eliz | Highly Engaged | Engaged after losing it for 1 week | Engaged |
| Luke | Highly Engaged | Disengaged | Engaged |
| Eva | Highly Engaged | Less engaged | Reengaged |
| Nate | Highly engaged/Motivated | Frustrated by daily goal |  |
| Sam | Engaged | Engaged | Disengaged after recent high engagement |
| Tristen | Highly Engaged | Very engaged |  |
| Broderick | Highly Engaged | Reengaged after disengagement | engaged |
| Wearability |  |  |  |
| Anna | Don’t notice it | Don’t notice it, part of day | Don’t notice it |
| John | Don’t notice it but don’t wear it to sleep | It’s just there | Don’t notice it |
| Matthew | Don’t feel it or are bothered but it’s ugly | Don’t notice it- but was wearing much less | Don’t notice it |
| Tom | Don’t notice it, like people asking about it | Don’t notice it | Don’t notice it |
| Mary | Notice it at night, like people asking about it | Sometimes bother (itchy), took off once | Like wearing a watch |
| Eliz | Don’t notice it | Don’t notice it | Don’t notice |
| Luke | Feels like watch | Conversation piece, feels like watch | Feels like a watch but don’t know if want to wear something on wrist |
| Eva | Don’t take it off | Don’t notice it | Don’t notice it |
| Nate | Don’t notice it | Don’t notice it | Sometimes itchy |
| Sam | Don’t notice it | Don’t notice at all | Didn’t find it a burden |
| Tristen | Like a watch | Easy enough | Uncomfortable |
| Broderick | Don’t notice | Notice the wearable | Don’t notice |
| Awareness |  |  |  |
| Anna | Like content but don’t read, look at progress multiple times per day | Not enough time for app messages, look at progress a few times per day | Like the messages, content, look in morning and evening |
| John | Addicted to updates | Notifications repetitive | Almost constant attention |
| Matthew | Checking progress constantly | Checking sometimes, no time for notifications | Checking periodically |
| Tom | Likes tips and checking progress | Enjoy reaching goal and checking occasionally | Quick look at status and content |
| Mary | It doesn’t ask a lot | Doesn’t capture my attention anymore – notifications repetitive | Notifications Repetitive |
| Eliz | Realize goals | Everyday is different | Morning and evening checking, no disruption during the day |
| Luke | Conscious I’m wearing it | Look at app less | Check the wristband but not the app |
| Eva | Doesn’t demand anything, check it more than Facebook | Doesn’t demand much attention at all |  |
| Nate | Checking automatically | Notifications don’t bother me |  |
| Sam | Advice quite fun | Look at it twice a day | Look at it twice a day |
| Tristen | Really interested in sleep | Shares with others | X |
| Broderick | Check every 30 minutes | Check 4 times per day | Check 2 times per day |
| The Embodied Wearable |  |  |  |
| Anna | Miss when it’s gone | Part of my body | Addictive. Looking for a watch with more functionality |
| John | Don’t notice it, just there | May keep just for training | Given running wearable as gift |
| Matthew | Didn’t miss it during a trip | Sleep suffering when not wearing | Need wearable to improve sleep. Looking for watch with more functionality |
| Tom | Crazy feeling when it’s off | Miss it when it’s off | Miss it when charging battery, looking for watch with more functionality |
| Mary | Comforted by sleep confirmation, felt like “missing something” when didn’t wear it | X | Had learned to tally steps in head |
| Eliz | Not something that I’m constantly affected by, but disappointed when don’t wear it | Feelings are confirmed, Couldn’t know the steps without it | Habit, feelings are confirmed, Looking for watch with more functionality |
| Luke | Never without it…Frustrated if couldn’t see progress | Could guess progress on own | Looking for watch with more functionality |
| Eva | I need to know, couldn’t know without it | I know steps without wearable | I need it, I don’t know the steps without it, doesn’t sleep well without it |
| Nate | Normal to wear it |  | Looking for watch with more functionality |
| Sam | I always have it on me but I’m not addicted to it, I couldn’t know the information without it | Questioning accuracy | I could know the information without it, but I prefer the technology. Looking for a watch with more functionality |
| Tristen | Felt bad after forgetting it | Missed it when loaned it to a friend for 1 night | Doesn’t want to know, has new baby and isn’t sleeping well, questions accuracy |
| Broderick | I know what kind of day I’ve had | The more you use it the more you gain, I miss it on the weekend when I’m active | Questions accuracy |
| Comparison to Another Device or Person |  |  |  |
| Anna | Similar to phone (check automatically) | Similar to app on iphone | Similar to first smart phone |
| John | A useful toy | Similar to smart phone | Smarter than smart phone |
| Matthew | Similar to smart phone | Calls notification ‘mother’ | Similar to first smart phone |
| Tom | Similar to smart phone (check automatically) | Similar to phone (check automatically) | Similar to phone (check automatically) |
| Mary | Similar to smart phone | Less use and less automatic than a smart phone | Part of your life, just like your phone |
| Eliz | Like your phone, always with you | More like your phone now that it’s automatic | More like your phone, automatic |
| Luke | Similar to favorite running app | Much less functionality than phone | Check automatically like a phone but like first blackberry |
| Eva | When sick it suggested to go for a walk and I felt much better | Check it like check phone | It could be like a phone if it started doing more |
| Nate | X | Maybe like a phone | Not like phone |
| Sam | Don’t use it automatically yet | Use it automatically like phone | Very different than the phone, use it much less |
| Tristen | Automatic like phone | Like the other apps I check every day | Similar to first smart phone |
| Broderick | 70% Similar to phone | If it had more functionality I would use similarly to my phone | Too little function to be like my phone |
